# Applying self-determination theory to behavior change technologies: insights from two projects in the health domain

**DOI:** 10.3389/fpsyg.2025.1634267

**Published:** 2025-11-25

**Authors:** Rebecca Gerstenberg, Emil Rosenlund Høeg, Jolene Van der Kaap-Deeder, Marc Hassenzahl

**Affiliations:** 1 Ubiquitous Design/Experience and Interaction, University of Siegen, Siegen, Germany; 2 Multisensory Experience Lab, Department of Architecture, Design and Media Technology, The Technical Faculty of IT and Design, Aalborg University, Copenhagen, Denmark; 3 Department of Psychology, Faculty of Social and Educational Sciences, Norwegian University of Science and Technology, Trondheim, Norway

**Keywords:** self-determination theory, health behavior change, autonomous motivation, reflection, rehabilitation

## Abstract

In the health domain, patients often need to adopt and maintain behaviors that are tedious, unpleasant, or even painful, such as taking medication regularly, being more physically active, or maintaining a diet. Behavior change technologies (BCTs) can play a crucial role in instilling change and maintaining healthy behaviors. One psychological theory to guide the design of BCTs is Self-Determination Theory (SDT). However, researchers within Human-Computer Interaction (HCI) struggle with its application, as the field lacks a shared understanding and reflection on the use of SDT. We present two HCI research projects in the health domain and reflect on the role SDT played in their implementation. Through a comparative analysis, we found that SDT was helpful in providing an analytical focus and guiding quantitative evaluations. However, its adaptation to qualitative methods and explicit design guidelines presents difficulties. Our reflection underscored the potential of SDT as both a theoretical lens and practical design tool for developing autonomy-supportive health technologies.

## 1 Introduction

The health domain is struggling with patient adherence to prescribed therapies (Celano et al., 2020; Enemchukwu et al., 2022; Glombiewski et al., 2012) and other forms of health-related behaviors, such as maintaining physical activity. A primary barrier is that these behaviors are rarely enjoyable in themselves, but can be rather tedious, unpleasant, or even painful, particularly during treatment and rehabilitation processes (Rice et al., 2019). Nevertheless, patients must adopt and maintain these behaviors for the sake of their health and recovery. Health behavior change technologies (BCT) have emerged as promising tools to optimize treatment outcomes and improve adherence, forming a distinct and growing domain within Human-Computer Interaction (HCI) (Aldenaini et al., 2020; Hekler et al., 2013).

Treatment adherence, understood as the long-term maintenance of recommended health behaviors, represents a complex biopsychosocial phenomenon shaped by interrelated biological, psychological, and social factors. While elements such as disease severity, family support, and socioeconomic status all play an important role, adherence is frequently attributed to the patient's motivation and self-efficacy. Consequently, research on BCT increasingly focuses on how to foster and sustain motivation to achieve sustained behavioral engagement.

One of the most influential theoretical frameworks for understanding motivation is Self-Determination Theory (SDT, Ryan and Deci, 2000), which posits that autonomous motivation, i.e., motivation that stems from personal volition and alignment with one's values, is the optimal driver of behavior (Cercos and Mueller, 2013; Tamborini et al., 2010). Substantial empirical evidence supports the beneficial effects of autonomous motivation on health-related behaviors (e.g. Ryan and Deci, 2017b; Wilson et al., 2006). In addition to improving performance quality, persistence, and well-being (Roth, 2019; Ryan and Deci, 2017b; Ntoumanis et al., 2021). However, much of this research has been conducted in human-to-human contexts, often neglecting a core aspect of HCI, the interaction with technology itself.

Although SDT has been increasingly adopted as a theoretical foundation within various HCI domains, such as games, health, education, and general behavior change, researchers and designers continue to face difficulties in applying SDT effectively in technological interventions. A review of how SDT has been applied in HCI games research revealed an overly descriptive and superficial use of the theory (Tyack and Mekler, 2020, 2024). Alberts et al. similarly observed that SDT is more often utilized to optimize user engagement with the technology, rather than to foster genuine behavior change itself (Alberts et al., 2024).

Despite SDT's strong empirical foundation and increasing use in HCI, there is a lack of systematic reflection and critical evaluation on how SDT is applied in technological interventions. To illustrate, Zhao and colleagues present a deep analysis of older adults use of technology for meaningful activities during the Covid-19 pandemic and derived design implications to enhance basic psychological need satisfaction but did not reflect on the theoretical implications of SDT in their approach (Zhao et al., 2023). In contrast, Aufheimer and colleagues provided a more integrated use of SDT by analyzing the work of physiotherapists through the lens of basic psychological need satisfaction and aligning it with strategies used in game design for physical therapy. Importantly, they discussed how SDT could serve as a framework for structuring therapeutic sessions, illustrating the potential for more systematic and theory-driven approaches to motivation in applied health contexts (Aufheimer et al., 2023).

Despite such promising examples, the lack of reflective, practice-based insights remains a major limitation in the field. There is a need for more retrospective analyses that examine how SDT is applied in design and evaluation processes, identify pitfalls and opportunities, and generate a shared understanding of best practices for integrating SDT into the design and evaluation of technological interventions. Researchers who have employed SDT in their projects are uniquely positioned to provide such reflections, offering both practical insights for technology design and conceptual feedback to SDT itself.

To address this gap, the present paper contributes a comparative analysis of two applied research projects in the health domain that utilized SDT as a guiding theoretical framework. Through this analysis, we aim to (1) examine how SDT has been applied in our technological health interventions, (2) identify opportunities and challenges in its practical use, and (3) propose directions for a more systematic and productive integration of SDT in HCI research. By doing so, this work seeks to foster a more critical and reflective dialogue on how motivational theory can inform the design of autonomy-supportive digital health technologies that enhance long-term treatment adherence and well-being.

## 2 Background

### 2.1 Self-determination theory

SDT is a comprehensive framework of human motivation, development, growth and well-being. It explores how social and contextual factors can either promote or hinder an individuals' sense of volition, motivation and psychological growth (Ryan and Deci, 2000, 2020). Developed by Edward Deci and Richard Ryan in the 1970s and 1980s (Vansteenkiste et al., 2010), SDT has become one of the most prominent and empirically supported theories of human motivation and well-being, explaining why people engage, participate, and exert effort in activities (Ryan and Deci, 2000, 2020) across various domains, such as education, work, parenting, health, and HCI (Ryan et al., 2006; Ntoumanis and Moller, 2023; Moller et al., 2023; van der Kaap-Deeder et al., 2017; Reeve and and, 2021; Tyack and Mekler, 2020).

**Figure 1:**
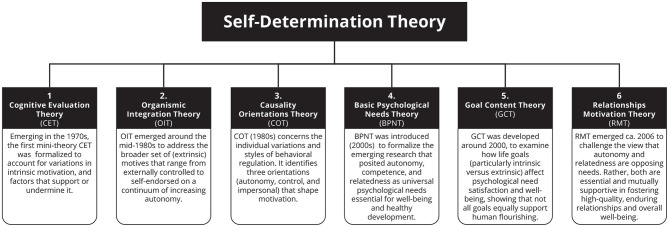
An overview of the six SDT mini-theories that make up the current SDT framework (based on Ryan and Deci (2019)).

SDT is structured into six interrelated *mini-theories* that together form an evolving macro-theoretical framework (Ryan and Deci, 2019, 2020). Vansteenkiste et al. compared this ongoing theoretical development with the assembly of a puzzle, with new conceptual pieces continually added through empirical research (Vansteenkiste et al., 2010). See Figure 1 for an overview of the different mini-theories of SDT.

At its core, SDT differentiates between autonomous and controlled motivation. Autonomous motivation refers to engaging in an activity out of personal choice and genuine interest, while controlled motivation is driven by internal or external pressures. The satisfaction of the three basic psychological needs—autonomy, competence, and relatedness—is central to fostering self-determined forms of motivation and psychological well-being (Ryan and Deci, 2017b). This distinction between autonomous and controlled motivation makes SDT particularly valuable for understanding how individuals adopt and maintain health-related behaviors and how technology can effectively support them.

#### 2.1.1 SDT and health behavior change

SDT has been widely applied in health care, as it represents a particularly valuable context to examine autonomy and adherence (Ryan and Deci, 2017a). A substantial body of research links SDT-based constructs—particularly autonomous motivation and perceived competence—to physical and psychological health outcomes (Ng et al., 2012; Ryan and Deci, 2017a). For instance, Williams et al. (1996) showed that autonomous regulation was a predictor of both exercise adherence and retention in a 6-month weight-loss program, with effects lasting beyond a year. Brunet et al. (2015) found that autonomous motivation was associated with higher levels of physical activity and positive affect, while controlled motivation was correlated with negative affect and depressive symptoms. Similarly, Segatto et al. (2013) observed that only autonomous forms of self-regulation predicted recovery-related physical activity, whereas external and introjected regulation did not.

Although such studies highlight the potential of SDT to enhance health motivation and outcomes, a recent meta-analysis noted that the effects of SDT-informed interventions on actual health behavior are modest and can be overestimated due to small-study bias (Ntoumanis et al., 2021). These interventions tend to show stronger short-term psychological benefits–such as increased motivation or need satisfaction–than long-term behavioral change (in follow up studies). These findings imply that, although interventions can demonstrate an increase in motivation, there is still a gap between psychological improvement and a lasting change in behavior.

To address this gap, researchers have begun to explore other SDT constructs such as internalization (Organismic Integration Theory, OIT), intrinsic aspiration (Goal Content Theory) and causality orientation to understand health-related lifestyles and risks in a wider context of aspirations and life values (Ryan and Deci, 2017a), see Figure 1. OIT offers a detailed account of extrinsic motivation and the process by which externally regulated behaviors can become internalized and self-endorsed (Ryan and Deci, 2017b; Høeg and Van der Kaap-Deeder, 2024). It distinguishes between external, introjected, identified and integrated forms of regulation, illustrating a continuum from controlled to autonomous motivation (Ryan and Deci, 2000). In health contexts where behaviors such as rehabilitation or treatment adherence are often externally prescribed and rarely intrinsically enjoyable (Eilert et al., 2020), OIT provides a particularly promising theoretical lens. It explains how such behaviors, though initially externally motivated, can become personally meaningful and autonomously maintained over time, making it highly relevant for the design of interventions seeking lasting behavior change.

#### 2.1.2 SDT and behavior change technologies

In parallel, digital technologies have become central to efforts to support and influence health behavior. In recent years, HCI researchers and industry developers have increasingly turned their attention toward creating systems explicitly aimed at shaping behavior (Rapp and Boldi, 2023; Alberts et al., 2024). These systems—commonly referred to as persuasive technologies (Fogg, 2002), BCT (Hekler et al., 2013), or mobile health (mHealth) (Molina et al., 2023) - employ interactive techniques such as reminders, rewards, and performance tracking to encourage or discourage specific behaviors (Rapp and Boldi, 2023). Exertion games (exergames) can also be considered a form of persuasive technology or BCT as they often seek to encourage and sustain physical activity through game-like features. However, for consistency, we adopt the term BCT in this article when collectively referring to these systems.

BCTs have demonstrated notable success (Hamari et al., 2014), for example, in avoiding smoking (Li et al., 2025), weight loss (Protano et al., 2024), and maintaining physical activity (Sullivan and Lachman, 2017). Yet, despite these short-term gains, a growing body of research has questioned whether technologies based on this approach can produce lasting behavior change and maintain user engagement over time (Rapp and Boldi, 2023; Alberts et al., 2024). Although SDT has occasionally been employed as a guiding theory in the design of BCTs, its use remains inconsistent. In their review, Alberts et al. (2024) found that only 15 of the 76 screened studies explicitly applied SDT, and many of these studies conflated constructs of CET and OIT, focusing predominantly on intrinsic motivation while neglecting the wider spectrum of extrinsic motivational processes (Alberts et al., 2024).

This oversight is notable given that OIT offers particular promise for BCT design. OIT's conceptualization of how extrinsic motivation can gradually internalize makes it uniquely suited for understanding and designing technologies that support long-term adherence. Alberts et al. (2024) identify two main SDT-based design approaches. We refer to these, following their reasoning, as engagement-focused and internalization-focused approaches.

(1) Engagement-focused approaches, aim to enhance intrinsic motivation and enjoyment by supporting autonomy, competence, and relatedness (typically based on CET and BPNT).
(2) Internalization-focused approaches, grounded in OIT, use technology as a scaffold for self-reflection value-alignment, helping users to transform externally motivated behaviors into self-determined ones. Engagement is not a goal in itself, and users are expected to become self-determined and less reliant on the technology.

Most current BCTs follow the first approach, emphasizing short-term engagement through gamification and feedback. However, Alberts et al. (2024) argue that internalization-focused approaches, although less common, are more likely to yield a sustainable change in health behavior. This highlights a major opportunity for integrating OIT more systematically into HCI and BCT design research.

### 2.2 Theoretical practices of SDT in HCI

Despite SDT's increasing application in HCI, its theoretical use remains scattered and conceptually fragmented (Tyack and Mekler, 2020; Ballou et al., 2022). Many studies adopt only selected constructs, such as intrinsic motivation, without engaging with SDT as an integrated framework. This selective use limits the field's ability to build cumulative knowledge and refine theoretical understanding.

Work in other theoretical traditions within HCI demonstrated the benefits of systematic synthesis. For instance, Clemmensen et al. (2016) developed a taxonomy of Activity Theory applications in HCI, clarifying how researchers employ the theory and identifying recurring interpretive challenges such as the use of selective constructs. Similar fragmentations have been observed in SDT-based HCI research (Tyack and Mekler, 2020). This suggests that comparable meta-level reflection is needed to consolidate and strenghten SDT's theoretical foundation within the field.

Following Hekler et al. (2013), we agree that researchers applying SDT are not merely users of theory, but contributors to its ongoing development. Each empirical application generates insights that collectively enrich the theoretical corpus of SDT-informed HCI research. Moreover, such reflection can identify conceptual tensions or ambiguities that warrant further theoretical elaboration. Bennett and Mekler's work on hedonic amotivation, which challenges the assumption that intrinsic motivation is universally positive, exemplifies how applied research can contribute to theoretical refinement (Bennett and Mekler, 2024).

Nevertheless, this kind of retrospective theorizing remains rare in HCI. As (van Berkel and Hornbæk 2023) argue, the advancement of theory in the field requires researchers to explicitly articulate the theoretical implications of their findings. In response to this call, the present work contributes a reflective analysis of the application of SDT in two healthcare projects. Through this, we aim to advance a shared understanding of best practices for integrating SDT, and particularly OIT, into the design of digital health technologies.

## 3 The application of SDT: two examples

To understand the challenges that must be overcome for a more fruitful application of SDT in HCI, and especially in technology for health, we present two projects that applied SDT. In the following sections, we ourselves will do a retrospective reflection on the use of SDT in our projects. We will elaborate on the role of SDT in specific steps of the projects and reflect on opportunities, as well as challenges that its application has brought to the projects. The first project (A) used SDT as a guiding theory in its design process, and in the second project (B), SDT-based measures were used to assess the project's goal of promoting intrinsic motivation. Although neither project exemplifies perfect integrated use of SDT, both illustrate a growing understanding and integration of SDT's theoretical concepts over the course of the project. In some cases, only subsequent reflection revealed the role of SDT in the specific project step. Thus, our insights on SDTs role were different during the projects themselves.

### 3.1 Project A: a persuasive environment for increased physical activity in knee osteoarthritis

#### 3.1.1 Aim and objectives—conceptually driven by SDT

**Figure 2:**
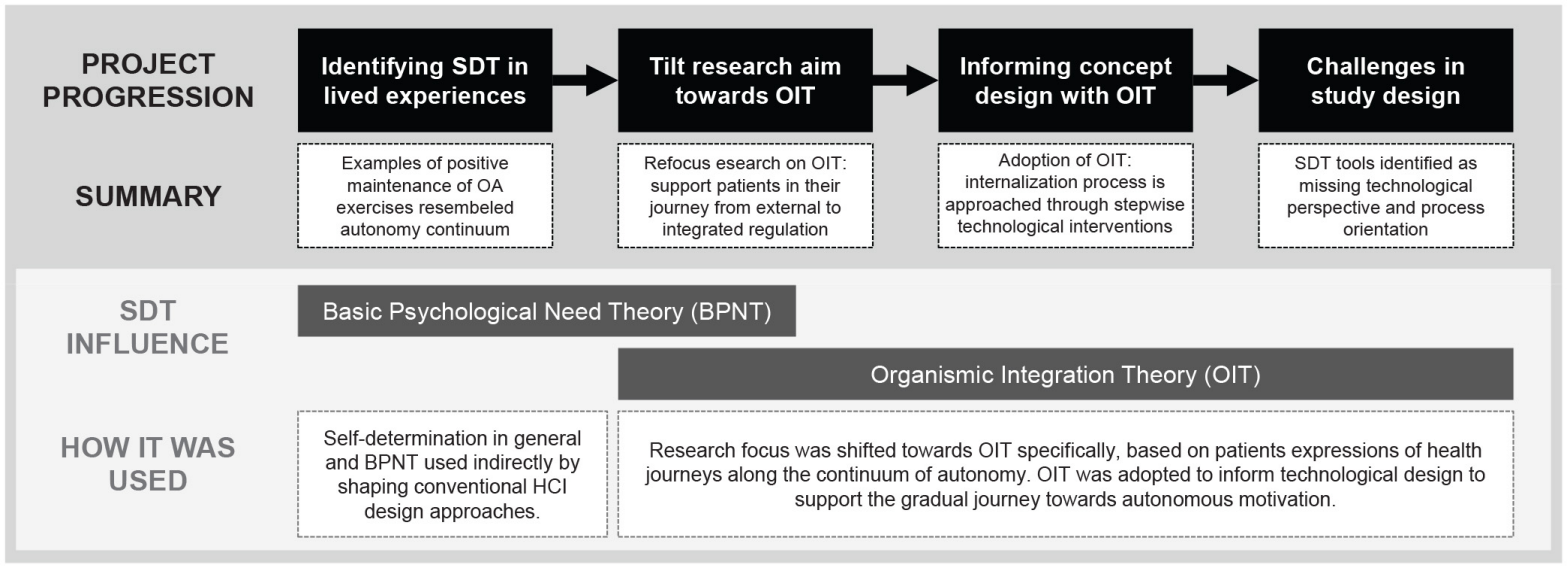
Project process of Project A and the role of SDT in its project steps.

Project A addressed helping knee osteoarthritis (OA) patients develop a joint-sparing and active daily routine to delay possible knee surgery. The emphasis was placed on maintaining physiotherapy exercises beyond the period of active therapy, as physical activity is a key therapy for OA (Rice et al., 2019). The goal was to design a persuasive environment (e.g. sensors, Internet of Things, app) for the everyday life of patients. SDT already played a role in the conceptual orientation of the project (Figure 2). For the design of the persuasive environment, interactive technology was thought to shape new healthy practices, which required a user-centered, sensitive strategy that explicitly seeks the balance between wellness and potential discomfort. We wanted the persuasive environment to respect and promote self-determination of patients so that they feel comfortable maintaining their health behaviors without feeling patronized by medical professionals or their illness.

#### 3.1.2 Exploration—Similarities in interview results and OIT

To create a better understanding of the situation of knee osteoarthritis patients, we conducted a qualitative interview study (Eilert et al., 2020). We aimed to determine how OA patients adopt and maintain beneficial physical activity practices during the course of their OA. The goal was to better understand successful motivational strategies in the real world. We recruited 10 “positive practitioners” (Klapperich et al., 2018) (7 women, 3 men; aged 52–76; median age: 58), people diagnosed with OA who believe they have developed an enjoyable, meaningful and sustainable practice of physical activity, to serve as inspiration for (re)designing the practices of “non-positive practitioners”. The recruitment was carried out through a German company with several physiotherapy and medical training centers in North-Rhine-Westphalia. Although methodologically, SDT did not play a significant role, as we asked for the “what”, “how” and “why” of a practice (Klapperich et al., 2019; Shove et al., 2012), we found that the journey these individuals underwent resembled the different types of regulation along the continuum of autonomy described by OIT (Ryan and Deci, 2017b). Our analysis revealed a journey of regaining autonomy and successful internalization - from initial loss of autonomy as a consequence of diagnosis, to controlled motivation through prescribed therapies and exercise regimens, and finally to a new state of autonomy with internalized routine physical activity practices.

#### 3.1.3 Design and development—Adapted OIT used as design approach

Recognizing the similarities with OIT during the interviews, we engaged more deeply with the theory. It advanced our knowledge of the motivating context in which patients find themselves when dealing with a chronic condition. This is why we sought to leverage OIT as a theoretical basis to help us create our subsequent persuasive environment. Not only because it validated our interview results, but also because it is argued that better use of behavioral and motivational theories as a basis for the design of BCTs will increase their effectiveness (Glanz and Bishop, 2010; Taylor et al., 2012). Thus, based on OIT, we wanted to gradually support and accompany patients in their journey from external to integrated regulation, thus fostering long-lasting physical activity practices. Although “autonomy support” has received broader attention in HCI (e.g., Deterding, 2016), we did not see the gradual journey we found in our interviews supported by designs for behavior change in HCI. Instead, joy, fun, and gamification predominate, along with other behavioral theories such as the transtheoretical model (Prochaska and Velicer, 1997; Prochaska and Diclemente, 2005) or the theory of planned behavior (Ajzen, 1991). Looking into SDT research helped to get inspiration (e.g. Hagger and Protogerou, 2020; Teixeira et al., 2020), but was not always easily transferable to technology design. For example, using the strategy “empathic listening” to support autonomy can be implemented in interpersonal but not technological interventions.

**Figure 3:**
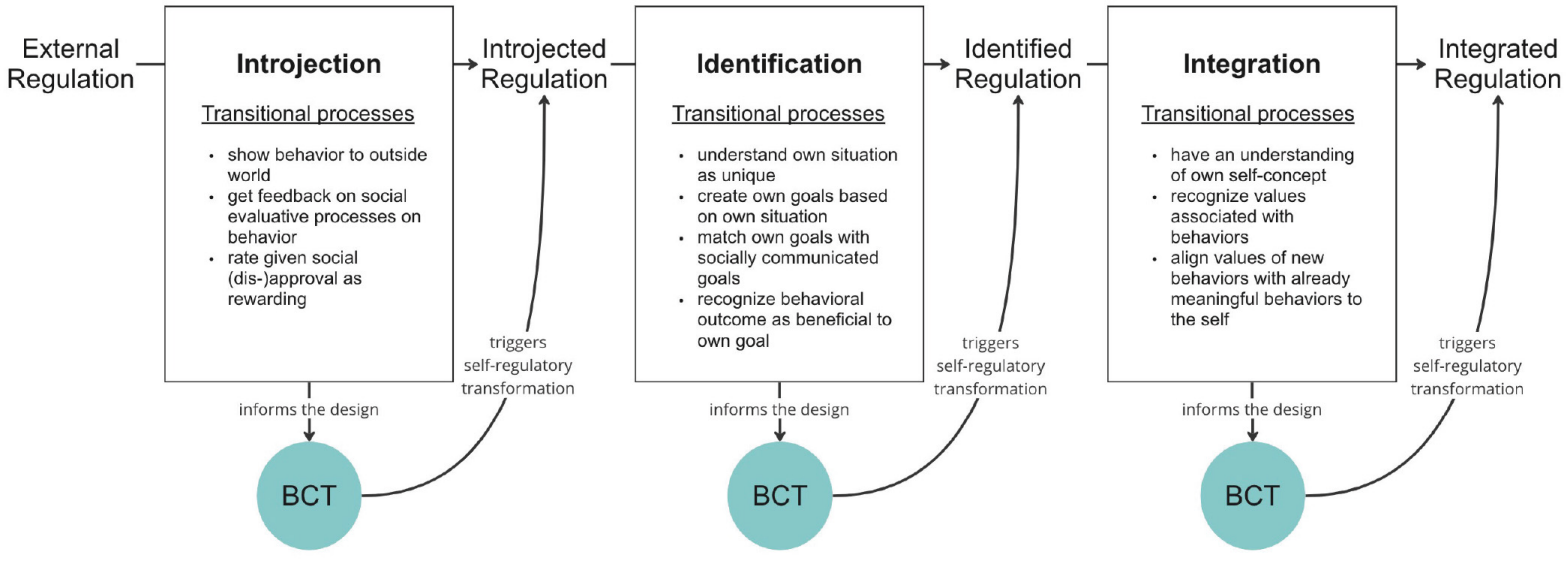
The framework of technology-mediated regulatory transformation: behavior change technologies (BCT) are positioned between regulation types to trigger self-regulatory transformation toward a more autonomous type of regulation. For more information see Gerstenberg et al. (2024).

In the end, we adjusted OIT by considering only one transition at a time to decompose OIT into a stepwise approach, instead of designing for the whole internalization process. Technology is thus positioned between the types of regulation as a transformational technology to facilitate the shift toward greater internalization (Gerstenberg et al., 2024). Even though this strategy is not fully consistent with the theoretical foundation as SDT does not view motivation in a stepwise fashion, it offered us the possibility of breaking down the complex internalization process into smaller units for design. Consequently, we developed concepts that each only targeted one transition, for instance, identification (introjected to identified) (see Gerstenberg et al., 2023 as an example for the transition of integration). Upon subsequently customizing them to a patient's position on their motivational journey; each technology shall facilitate greater internalization from this point forward. Consequently, the internalization process is approached through stepwise technological interventions. Figure 3 illustrates this theoretical adaptation of OIT to the Framework of Technology-Mediated Regulatory Transformation.

In summary, although OIT guided our design approach, its abstract nature made it difficult to apply directly to specific design decisions. It remains rather vague in its descriptions of the psychological mechanism of different types of regulation. Open questions remain, for instance, what will a person do to internalize a behavior and what happens during a change of regulation types?

#### 3.1.4 Evaluation and findings—In search for tools to customize according to OIT

**Figure 4:**
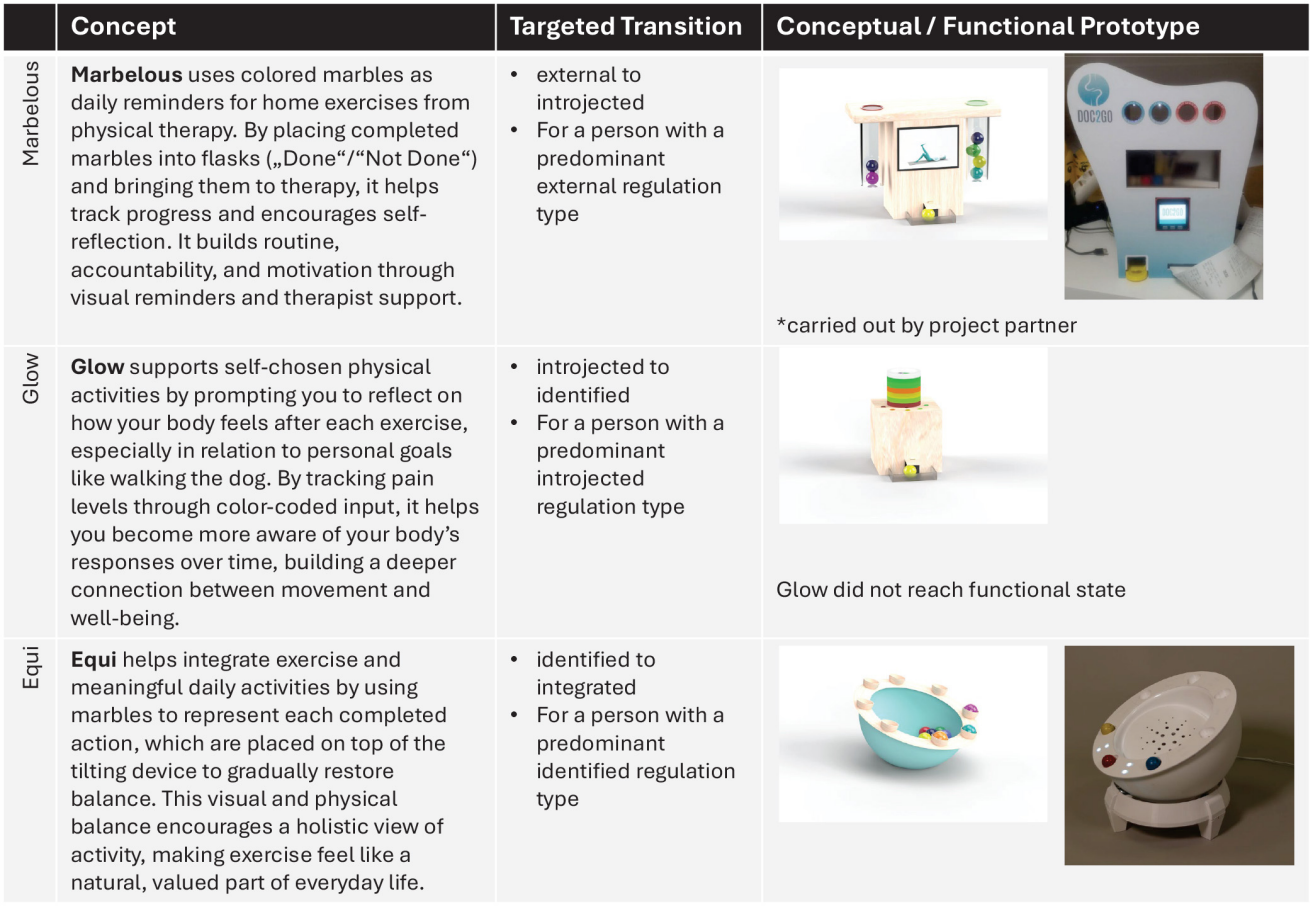
Overview of the prototypes developed in Project A. This overview was inspired by the design proposals outlined in Şener and Pedgley (2023).

In light of this design approach and as a means of exploring whether and how our concepts can help patients with OA become more autonomous in their physical activity practices, we conducted exploratory field studies with the different transitional interactive prototypes we created (see Figure 4 for an overview). Thus, we needed a way to classify the participant's positions in their OA journey to offer them the appropriate prototype. Our idea was to assign patients a “predominant” regulatory type to give them the design that targeted the transition that follows their predominant regulatory type. However, the concept of a predominant regulation type is a new idea, and thus there is no instrument for this purpose yet. Therefore, we looked for scales that measure the different forms of regulation, such as the Behavioural Regulations in Exercise Questionnaire (BREQ3, Wilson et al., 2006). BREQ3 focuses on behavioral regulation in physical activity, but has not been used to define a “predominant” type of regulation. Therefore, we used BREQ3 to calculate the Relative Autonomy Index (RAI), a single score calculated by weighting and summing the subscale scores, providing an index of the extent to which respondents feel self-determined. We adapted the RAI insofar that we divided it into six equal-sized sections for the six types of regulation (amotivation to intrinsic). We then assign the participants to the type of regulation according to their RAI. For example, when the range of RAI would be from -24 to +24, a person with a RAI between -24 and -16 would be assigned to a predominant external regulation type, a person with a RAI between -16 to -8 would be assigned a predominant introjected regulation type, and so on. On this basis, we distributed the participants to the different field studies. To identify a change in the RAI, and thus the predominant regulation type, participants also answered the BREQ3 after the field study. However, since the studies used relatively small sample sizes (e.g. 5 participants), the results could often only be indicative.

### 3.2 Project B: investigating the motivating capabilities of virtual reality-based rehabilitation

#### 3.2.1 Aim and objectives

**Figure 5:**
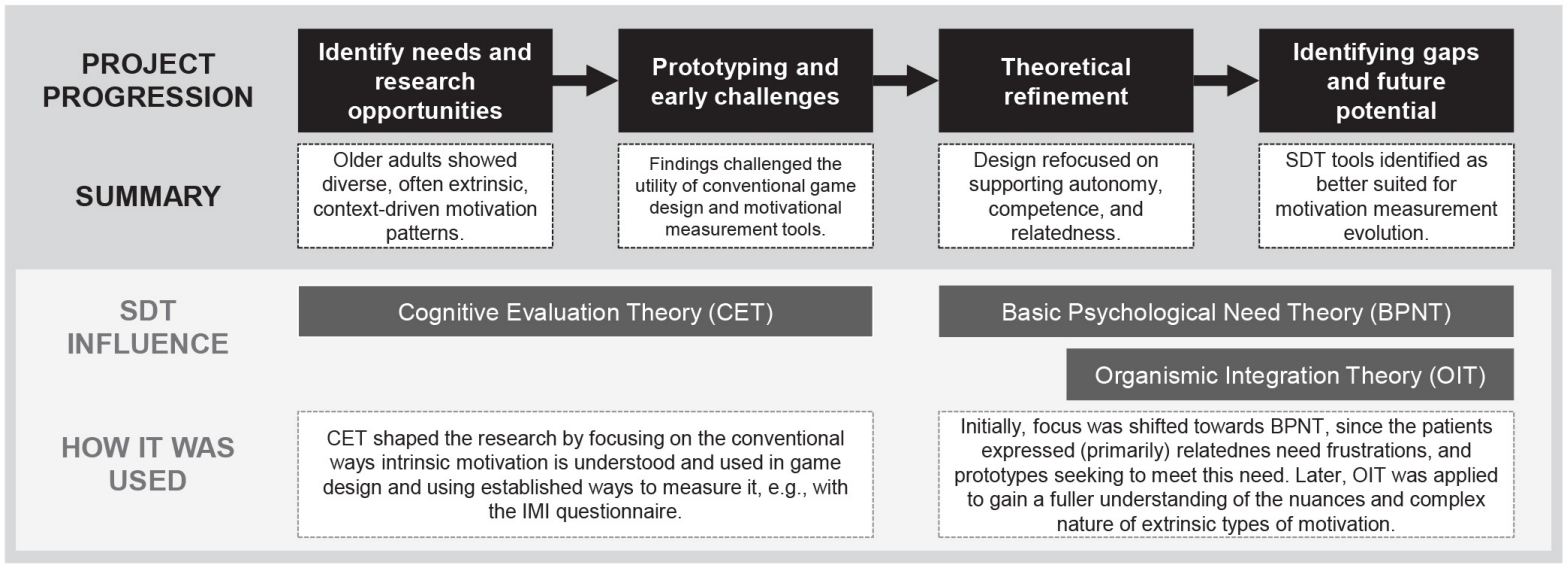
The project process of Project B and the role of SDT in its project steps.

Project B explored how virtual reality (VR)-based exertion games (exergames) combined with cycle ergometers might cultivate intrinsic motivation for physical activity among older adults undergoing rehabilitative treatment in two municipal facilities (Høeg et al., 2020b). The project followed another completed research project on VR-based exercise in nursing homes with a similar scope of encouraging physical activity among older residents who were often not very motivated to engage and persist in physical activity (Bruun-Pedersen et al., 2014). The project stakeholders designed, developed, evaluated and implemented a specific bike-based exergame solution to encourage physical activity among the elderly, with very promising results (Bruun-Pedersen et al., 2014, 2016b,a) This project sought to continue the research in two new and different contexts: a) a municipal Outpatient Health Centre (OHC) and an Inpatient Rehabilitation Unit (IRU). The scope of the project was similar, i.e., to encourage physical activity and persistence among patient populations who often had low adherence. However, the scope was also more pluralistic, as it sought to investigate new design opportunities to improve the effectiveness of the VR-based physiotherapeutic treatment applications. In addition, our goal was also to design, redesign, and develop new bespoke systems optimized for therapists (primary user) and patients (secondary users) to be implemented as a practical tool that therapists could utilize to provide more effective and (intrinsically) motivating treatment (Høeg et al., 2020b). As such, until gaining a fuller understanding of SDT, the VR exergame was initially viewed as an instrument that created a shift in motivation from extrinsic toward intrinsic motivation. The initial goals of Project B were to explore new design opportunities and to improve the design of the VR exergame for the purpose of increasing motivation. See Figure 5 for a visualization of the project process.

#### 3.2.2 Exploration

The project adopted a pragmatic research approach, which integrates the view that neither subject nor worldly phenomena can be understood without considering the context (Creswell and Poth, 2017). Therefore, thoughts, actions, objects, and events must always be viewed in the context of the broader situation, and that includes what motivates the individual. Pragmatism encourages the involvement of the researcher and allows subjectivity, particularly when making conclusions based on the experiences and behaviors of the participants (Creswell and Poth, 2017). The research approach involved concrete methods to unravel the experiences of the therapists (the primary users) and the patients (the secondary users). These methods involved ethnographic observations, participatory design (co-design), autoethnography, and contextual inquiry (Viitanen, 2010). More specifically, the project utilized a contextual design approach (Beyer and Holtzblatt, 1999), conducting research in the two rehabilitation facilities, to explore the interactions with the implemented systems in an applied context (Høeg et al., 2020b).

#### 3.2.3 Design and development

**Figure 6:**
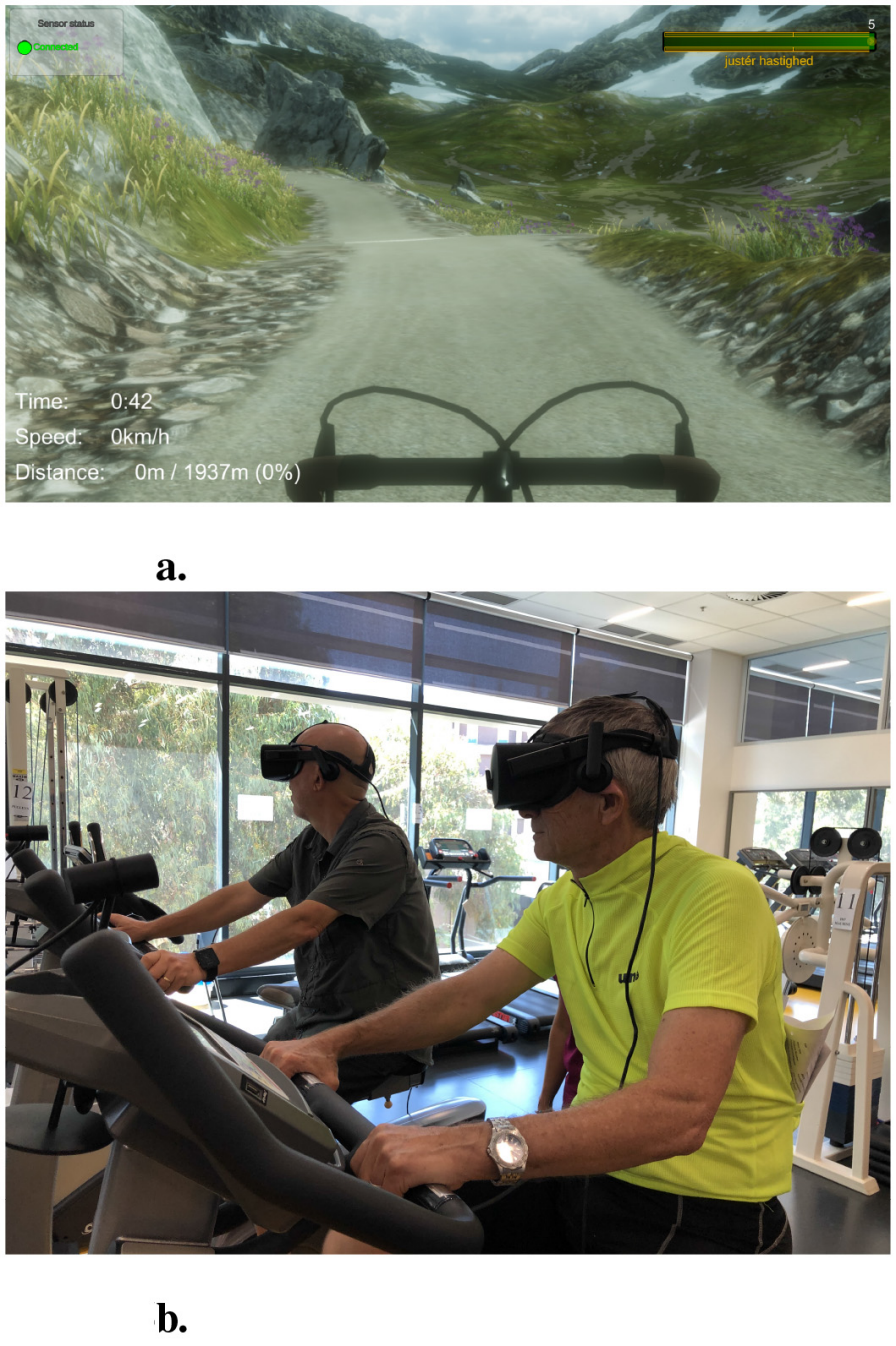
The basic recurrent design of the “VR Bike” included a VR environment **(a)**, an exercise bike with a cadence sensor. In the “Buddy Biking” experience, users **(b)** would exercise together on separate physical bikes while also being co-present on a virtual *tandem bike*, to support relatedness. See Høeg et al. (2021b) for more details.

New prototype concepts were explored through multiple workshops with therapists, to ideate and design new prototypes that converged the needs of both primary and secondary users (Høeg et al., 2020a). The insights gained from this approach led to prototyping and research projects that sought to increase performance in the local Chronic obstructive pulmonary disease (COPD) program in the outpatient facility (Høeg et al., 2021a). To measure the older adults motivation, the intrinsic motivation inventory (IMI) questionnaire was initially used to verify whether the solutions were capable of instilling a shift in motivation. Subsequently, the interviews and workshop findings allowed a more nuanced understanding of motivation, based on the diverse expression of needs and frustrations. This led to the adoption of the Basic Psychological needs theory (BPNT) mini-theory (Ryan and Deci, 2000). Later, the OIT was also adopted to explicate the nuances of motivation (Ryan and Deci, 2000). This led to a more focused effort to create prototypes that supported the expressed needs of the patients, which was largely focused on the need to socialize with fellow inpatients (Høeg et al., 2021b, 2023). See Figure 6 which illustrates the *Buddy Biking* system.

#### 3.2.4 Evaluation and findings

The observations and interviews with the older adults revealed diverse motives and perspectives that explicated their adherence to therapy. The participants articulated a range of reasons, including a general aversion to physical activity (e.g., “it is boring”) and negative perceptions of the inpatient environment (e.g., “it is a sad place to be in”), both of which appeared to impact their engagement with the various rehabilitation programs. This highlighted that participation in VR therapy was not always rooted in intrinsic motives. Moreover, further inquiries unearthed several potential design suggestions that did not rely on traditional game design principles, but rather on a propensity for nature exploration and the need for greater social interaction with other patients and caregivers. Although participants generally expressed situational enjoyment and satisfaction from interacting with the exergame, we did not really have adequate baseline measurements to compare the findings. Since the studies used relatively small sample sizes and non-comparative study design, the IMI could often only be used as an indicative result. Any variation of the technology or non-technological intervention could likely have given similar results, i.e., without a comparator, the IMI, in this exploratory approach was not particularly useful on its own. This realization was further emphasized by the many different reasons why many older adults did not participate or persist in the offered rehabilitation programs. In applied research, which is highly encouraged in implementation research, the context of the situation is often paramount to consider, especially since many factors can influence project results. For instance, how much sleep did the participant get? How is the participant currently coping with these new life-changing circumstances (e.g. loss of function)? What is the impact of the motivation of the primary user (therapist) to use VR with the patients? These emerging questions led to a more thorough perusal of SDT literature, which led the project to adopt both basic psychological needs into the design and a richer understanding of the different controlling and autonomous types of motivation (OIT).

In this new view of motivation, we started asking the question: *how can the exergame create the right conditions for the patients to experience autonomous motivation?* rather than *how do we create an exergame that motivates intrinsically?* And, through the SDT literature, we started more top-down with how the technology could support the experiences of autonomy, competence and relatedness and thereby fostering (internalized) motivation. However, we still needed a suitable instrument to measure the effect of introducing such an experience into the rehabilitation phase, both for primary and secondary users. One that could measure not only intrinsic motivation, but other types of motivation as well, and preferably in an instrument that could compare standard therapy with the new intervention.

If patients are referred for treatment and given the opportunity to engage in VR-based therapy, the experience is likely to provide them with a sense of satisfaction and (hedonic) enjoyment from otherwise monotonous exercises and meticulously planned inpatient activities, which can be expressed as intrinsic motivation. However, while BCT can potentially result in an improvement in motivational quality, other environmental and personal factors may influence their general well-being and motivation negatively (e.g. lack of sleep, psychological needs thwarting, coping difficulties). Additionally, it is equally important to consider the motivational regulation of the primary users and how this relates to the propensity to offer treatment and what qualities of the experience are communicated to the secondary user. Therefore, we stipulate that there is much to gain from adopting SDT instruments that measure the wider continuum of motivation rather than only intrinsic motivation. Unfortunately, the project was already well advanced and was largely obstructed by the Covid-19 pandemic, and we did not have the opportunity to apply these new insights. However, the project sparked further investigations into how other SDT-based measures may be more appropriate to help grasp different types of regulation and their change due to the interaction with technological intervention.

## 4 Reflection on the use of SDT

Comparing the use of SDT in both projects, you can see that the two projects employed SDT in different ways. However, commonalities can still be identified. While project A aimed to incorporate OIT into the design process, project B focused on intrinsic motivation. For this, both projects needed to create new ways, either a theoretical reduction or measures, to adapt SDT to HCI work. The findings have also been summarized in Table 1.

**Table 1:** Summary of *findings* and *lessons learned* based on the identified themes explicated from the two projects.

**Theme**	**Findings**	**Lessons learned**
**1. Theoretical focus and translational challenges**	• The choice of SDT mini-theory determines the research focus • Intrinsic motivation may not reflect actual user motivation, which is often more complex. A singular focus on intrinsic motivation may overlook other relevant motivational factors.	• Choose SDT mini-theories (e.g., OIT) that align with the motivational context. • Recognize types of extrinsic motivation as valid. • Make transitions and coexistence of regulation types explicit in design.
**2. Different sources of motivation**	• The motivation for using technology and the motivation for the target behavior (e.g., exercise regimes) can diverge.	• It is important to distinguish between these manifestations of motivations and needs while also assessing their interplay. • Technology disengagement is not a failure, but possibly a sign of user autonomy.
**3. Adaptation of metrics and methodological origins**	• HCI's qualitative approach contrasts with SDT's quantitative tradition. • Existing SDT tools (e.g., BREQ3, IMI) had limited applicability when measuring technology in context adaptation • Adaptation is necessary, but often insufficient, and also risks compromising validity	• Assess not just motivational constructs but also technology-mediated need fulfillment. • Utilize or adapt instruments specific to technology-mediated motivation (e.g., based on the User Motivation Inventory Brühlmann et al., 2018).
**4. Lack of design guidelines**	• Technology use in real-world environments is complex and organic. • There are insufficient design techniques to accommodate multiple motivational states. • It is challenging to adjust technology to dynamically changing user motivation.	• Investigate design strategies rooted in OIT to support progression toward autonomous motivation. • Future systems may seek to be more adaptive to the users dynamic motivational states.

### 4.1 Theoretical focus and translational challenges

In contrast to Project A, which applied OIT conceptually, Project B focused more in intrinsic motivation and practical implementation within a clinical context. Similar to what Jansen and colleagues (Jansen et al., 2017) found when applying the three basic psychological needs to persona creation, the choice of (mini-)theory determined the analytical focus in our projects. Although this allows for a deep focus, it can lead to neglecting other areas that could also be valuable. For example, participants in Project B reported experiencing autonomous motivation due to the interaction with the technologies developed that are not yet intrinsic. This created friction in the ongoing research foci. Whereas in Project A the theoretical basis was chosen from the results of the explorative interview study, the focus on OIT might have hindered an easier translation into technological design. For example, SDT research suggests to fulfill the basic psychological needs in order to pursue autonomous motivation (Gillison et al., 2019). Translational research in HCI for need-fulfilment is already available, which could have simplified the design process (Cherubini et al., 2020; Hassenzahl, 2010; Hassenzahl et al., 2012; Villalobos-Zúñiga and Cherubini, 2020; Villalobos-Zúñiga et al., 2021).

Since the chosen theory influences the analytical focus, it should reflect the motivational context in which it is actually being studied. For example, OIT emerged in both projects from the specific motivational context in which intrinsic motivation did not match participants' experiences and reflections on their health practices (either a-priori or a-posteriori of technological developments). Future research should match their motivational context in question to the best possible (mini-)theory of SDT. This means that first of all we need to identify the motivational context we aim to investigate. For example: *Do I want to help people get motivated at all (overcome amotivation)? Do I want to help people make long lasting or short-term changes (controlled or autonomous motivation)? Can I help people achieve intrinsic motivation or will there always be an external reason for their behavior (extrinsic vs. intrinsic motivation)?* Intrinsic motivation might be considered the “ideal” motivation, but there are contexts in which it is hard to achieve. Thus, we need to overcome the dichotomy of extrinsic versus intrinsic motivation in favor of a more general exploration of extrinsic motivation, goal contents, or causality orientations.

Both projects were conducted in the context of health, with OIT aligning particularly well with this context for several reasons. First, patients with (chronic) health problems must maintain preventive behaviors that they most likely do not find pleasant. Second, the impending health condition itself is a form of extrinsic motivation that is already present. Third, a worsening condition or physician recommendations for a behavior, e.g., more physical and less sedentary activity, are examples of extrinsic demands that must be internalized in order to feel better again. In this context, a focus on extrinsic motivation seems appropriate. However, as our projects show, some obstacles must first be overcome for OIT to become fruitful for technology design and evaluation. Its potential lies within its diversity and procedural approach of controlled to autonomous motivation, which can provide new insights into why and how BCTs work. Especially Project A shows that a deeper and more explicit understanding of relevant constructs of OIT, such as how people can achieve specific regulation types, is highly needed from SDT-research. Such insights into how motivation evolves toward being more autonomous can further yield strategies for design and evaluation.

### 4.2 Adaptation of metrics and methodological origins

SDT-research already presents a plethora of metrics and methods to assess related constructs. The official website of the Center for Self-Determination Theory lists 37 independent instruments (as of May, 2025). However, only one, the Autonomy and Competence in Technology Adoption Questionnaire (ACTA) (Peters et al., 2018) is concerned with the use of technology. Both projects tried to integrate validated tools from SDT-research into their studies (BREQ3 in Project A, IMI in Project B), but struggled for various reasons. Due to a lack of questionnaires related to the use of technology, we needed to adapt existing ones to our study context. We acknowledge that this must occur to some extent in any study. For example, in Project A we studied physical therapy home exercises to help the patient with OA, which is why we adjusted the wording of the BREQ3 (“exercise”), to be targeted toward specific OA exercises (“osteoarthritis oriented home exercises”). However, due to the psychological origin of the metrics, they do not take into account the unique situation of technological interventions. For example, in Project B the participants wanted the technology to help with greater social interactions with other patients in the form of collaboration. Although IMI could be used to measure whether the relatedness has increased in its current form, it cannot be used to measure whether the use of VR technology mediated this experience of relatedness.

We suggest that HCI researchers who want to use SDT in the future assess whether the technological intervention changes the motivational constructs we want to measure and whether SDT metrics (with a little adaptation) can measure the constructs in question. For example: *Do we want to measure the motivation to use the technology we developed? Do we want to measure whether the need for (interpersonal) relatedness has increased in users, whether the technology mediated this need-fulfillment, or whether the participants feel related to the technology itself?* In either way, there is a need in HCI to tailor metrics toward particular technological applications in interventions. The User Motivation Inventory by Brühlmann and colleagues (Brühlmann et al., 2018) is a good example for this.

The different methodological origins of HCI and SDT research were another reason we struggled to find the right tools for our projects. Both projects had small sample sizes due to the (qualitative) methodology used, such as contextual inquiry or case studies. HCI has a long history of using qualitative methods to examine the experience created through technologies that we develop. For example, Project A wanted to explore how technological interventions influenced the experience of self-regulation and any changes to it. Project B wanted to disentangle users' experiences with a new VR-based physiotherapeutic treatment in a clinical setting. Supplementary, both projects added SDT-metrics to measure self-regulation types (Project A) and intrinsic motivation (Project B). As a result of the small sample sizes, their results could only be used indicatively and had no statistical relevance.

SDT, on the other hand, with its psychological background, has a long history of using quantitative methods to evaluate hypotheses. Qualitative methods are rather used with restraint. Future efforts of HCI researchers to use SDT must take this into account when considering the use of SDT tools, as qualitative and quantitative methods ask different questions. Where SDT wants to understand what social conditions thwart or support human flourishing, in HCI we often want to understand how we can apply the social conditions found in SDT to interactive systems in order to support human flourishing technologically. The METUX model by Peters et al. (2018) is an approach to fill this gap; however, its evaluation suggestions are strongly influenced by the quantitative methodological background of psychological studies. Other options include integrating validated SDT tools where the research question and method(s) match (e.g., Molina et al., 2023), but also to further integrate SDT constructs into common HCI research methods, such as interviews, to enrich the constructs with qualitative descriptions (e.g., Deterding, 2016). For example, Project A tried to define a predominant regulation type through the BREQ3-questionnaire, which has not been used like this before. Another possible solution might be to conduct interviews to define a predominant regulation type, which could also reveal information on when, why, and how different regulation types coexist. In this way, HCI researchers could also contribute to the development and refinement of SDT (Hekler et al., 2013) and gather explicit design implications, for example, for the design of autonomy.

### 4.3 Different sources of motivation

Both technological interventions in the described projects had the goal of changing the quality of motivation for health behaviors, addressing two questions regarding motivation. A) *do users have the motivation to use the developed technologies?*, and B) *(how) does motivation toward the health behavior change over time?* The projects show that these questions are not easy to distinguish from each other. For example, Project B found that participants enjoyed the VR treatment and had fun. They concluded that patients who are introduced to health games as part of their treatment may find enjoyment in the interaction (with VR), but it is unlikely that the health activity is something they would pursue volitionally outside of therapy, i.e., intrinsic motivation toward the health behavior was ultimately not cultivated. Furthermore, they suspected that their “intervention” will most likely always yield higher intrinsic motivation than standard care because of its newness and playfulness and questioned whether that is even a result on its own merit. It would be valuable to explore whether a higher quality of motivation in one area (e.g., engaging with VR) could positively influence motivation in another area (e.g., adopting health behaviors and or increasing general well-being). This also raises the question of how best to assess what parts of our technological interventions are the reasons for a motivational change in behavior.

The Project A collaborators did not find a change in the users' particular types of self-regulation. Nevertheless, they found participants expressed different motivation types depending on the source they discussed. When referring to their motivation toward the behavior itself, participants made statements typified as autonomous regulation. In contrast, their motivation toward the technology was mostly characterized as controlled and externally driven. This opened the discussion on whether the two types of motivation must coincide or whether they can diverge, that is, if external interventions can prompt people to become more autonomous. Since SDT proposes that external motivation can undermine autonomous motivation, this is an important question with regard to technological interventions. Project A proposes it to be a delicate balance, but it is possible to become more self-determined by being other-directed. This distinction between motivation derived from technology use and motivation toward a specific behavior is unique to the application of SDT to technological interventions and, as such, to the use of SDT in HCI. Consequently, it posits possible future endeavors for researchers translating theory into practice.

### 4.4 Lack of design guidelines

Most of the difficulties that the projects faced in using SDT were related to their design processes. The design approach of Project A was based on OIT, which made it necessary to understand the users' changing motivational state, i.e., the transitional processes between types of self-regulation. SDT posits that these different types of self-regulation exist and that they differ from each other in their fulfillment of autonomous functioning (Ryan and Deci, 2017b). However, it remains rather vague in its description of what is happening in a person during a transition of self-regulation. Since this approach is not widespread, there is a dearth of research concerning design techniques for self-regulatory transitions. For technology to be customized to the changing motivational state of a user, the transitional process needs to be made more applicable. Although interpersonal interventions adapt implicitly, for technological design, this needs to be made explicit, e.g., *how can technology recognize in what motivational phase a person is, since it is ever-changing and dependent on the social context? How can we adjust the technology to changing states of motivational regulation? How can we design technology for several motivational states simultaneously since SDT states that regulation types can coexist?*

Technological design requires clearer design guidelines. For example, future systems should be able to recognize and adapt to diverse motivational contexts based on user interactions. But first, we need to understand how we can define a person's motivational state through their actions. For example, it is often criticized that BCTs are abandoned quite quickly, and consequently discussions are centered on how to maintain engagement and retain use (Fritz et al., 2014; Karapanos, 2015). However, abandonment of technology might also be a sign of autonomous motivation, where external support is not needed anymore, or even stands in the way of a fully autonomous experience. Thus, we need to question the paradigm that only sustained engagement with a technology is what creates motivation (Alberts et al., 2024).

## 5 Discussion

Despite the different orientations and implementations of the projects, we found several consistent theoretical implications in our comparative analysis of the application of SDT in two healthcare projects. For example, both projects found that OIT is a mini-theory that seems particularly appropriate for the health domain. In contrast, supporting intrinsic motivation in behaviors that are not self-selected but imposed by illness, and often not pleasant, does not seem to fit well here (different from games research). Furthermore, we encountered typical pitfalls that HCI researchers should be aware of when integrating SDT theory into their work. For example, being aware of the different methodological origin and history the field of psychology has. This field largely does not deal with the unique role the interaction with technology brings into human life, but rather tries to understand individual functioning in the world. In addition, theories are only an abstracted model of reality. When we investigate the specific context and real-world usage of our technological interventions, we need to accept that the theory might not always fit perfectly to this setting. Consequently, models, tools, and guidelines need to be adopted in the specific context of human-computer interaction. This also offers the possibility to question the theoretical assumptions made by SDT. For example, Bennet and Mekler challenge the purely positive connotation of intrinsic motivation (Bennett and Mekler, 2024).

In Project B, the users requested that the technology supported their need for relatedness to other inpatients. This raises the question whether the relationship that users form with the technology might also cater to the need of relatedness. This example illustrates how reflecting on the application of SDT can help researchers identify where theoretical assumptions and constructs are too immature in predicting outcomes and thus in designing technologies. Furthermore, this reflective process can help to formulate new questions that are specific to technological interventions, e.g., concerning the motivational source or the influence of autonomous motivation on technology engagement. As van Berkel and Hornbæk propose, reflection on the use of SDT in the two projects improved our understanding of the limitations and possibilities of SDT in technological research (van Berkel and Hornbæk, 2023). For example, we saw that SDT might not be able to fully account for some technological phenomena (e.g., intrinsic motivation due to novelty effect), how to apply theory to more cases (adopt SDT tools to qualitative cases), or see new mechanism drive technological research (autonomous motivation might alter our understanding of successful technological adoption). We believe that this process of self-reflection on theory use offers beneficial insights to generate an overview and shared understanding of the application of SDT in technological research. However, we have devoted the entire paper to this reflection process in order to obtain theoretical implications. We understand that researchers who add a section on theoretical implications to their “regular” articles may need to summarize and shorten their accounts of their application of SDT.

Above all, this careful reflection on the use of SDT in technological research shows a fundamental aspect. To overcome the shallow and descriptive use of SDT in technological design (Tyack and Mekler, 2020, 2024), one should not use SDT as an unquestioned theoretical basis for technology design. Researchers seeking to ground technological developments in a strong theoretical foundation need to ask questions such as which mini-theory or construct of SDT really represents their ideas theoretically. In this work, we provide some questions that researchers who want to apply SDT in their technological projects could ask themselves before relying on it as a theoretical basis.

## 6 Limitations

Although our goal is to contribute practice-oriented insights into how SDT can inform the design of digital health technologies within HCI, we also recognize the limitations of our work. Our reflections are based on two projects and thus offer a limited scope for generalization. Nevertheless, we believe that these reflections can support other researchers who use—or intend to use—SDT as a theoretical foundation. Our findings may help them compare their own experiences with ours or encourage them to consider similar aspects in their design processes. We do not present our conclusions as definitive truths, but as an invitation to dialogue: a first step toward developing shared best practices for applying SDT in digital health design. Ultimately, our work aims to spark more integrated, theory-driven research that continues to build this collective understanding.

## 7 Conclusion

Our paper reflected on the use of SDT in two projects in the context of health, which investigated how to motivate sustained behavior change. To this end, we have taken a closer look at the role of SDT in each project step and reflected on the opportunities and challenges. Despite the divergent approaches, the comparison shows that the chosen theoretical focus determined the analytical process of the projects. Although remaining within a single theoretical perspective can sometimes be beneficial, in the context of health, we found that this approach may overlook other important factors. To gain a more pluralistic understanding of the context, the mini-theory of OIT proved particularly useful. It helped us expand the design space to better support autonomous motivation, which is especially valuable in areas where fostering intrinsic motivation can be challenging. Furthermore, our SDT-based technological interventions showed unique obstacles in translating insights from human-to-human interventions. Experience-based differences between human-human interaction and human-computer interaction must be considered to successfully implement SDT in technological research. We encourage researchers who apply SDT in their work to reflect on its role in their research and to share the resulting theoretical insights in their publications. Doing so can help build a more cohesive and shared understanding of SDT across various research contexts.

## Data Availability

The original contributions presented in the study are included in the article/supplementary material, further inquiries can be directed to the corresponding author.
